# MR-based follow-up after brachytherapy and proton beam therapy in uveal melanoma

**DOI:** 10.1007/s00234-023-03166-1

**Published:** 2023-05-30

**Authors:** Michael C. Y. Tang, Teresa A. Ferreira, Marina Marinkovic, Myriam G. Jaarsma-Coes, Lisa Klaassen, T. H. Khanh Vu, Carien L. Creutzberg, Myra F. Rodrigues, Nanda Horeweg, Yvonne L. B. Klaver, Coen R. N. Rasch, Gre P. M. Luyten, Jan-Willem M. Beenakker

**Affiliations:** 1 Department of Ophthalmology, Leiden University Medical Center, P.O. 9600, 2300 RC Leiden, The Netherlands; 2 Department of Radiology, Leiden University Medical Center, Leiden, Netherlands; 3 Department of Radiation Oncology, Leiden University Medical Center, Leiden, Netherlands; 4 Holland Proton Therapy Center, Delft, Netherlands

**Keywords:** Uveal melanoma, Magnetic resonance imaging, Brachytherapy, Proton beam therapy, Ultrasound

## Abstract

**Purpose:**

MRI is increasingly used in the diagnosis and therapy planning of uveal melanoma (UM). In this prospective cohort study, we assessed the radiological characteristics, in terms of anatomical and functional imaging, of UM after ruthenium-106 plaque brachytherapy or proton beam therapy (PBT) and compared them to conventional ultrasound.

**Methods:**

Twenty-six UM patients were evaluated before and 3, 6 and 12 months after brachytherapy (*n* = 13) or PBT (*n* = 13). Tumour prominences were compared between ultrasound and MRI. On diffusion-weighted imaging, the apparent diffusion value (ADC), and on perfusion-weighted imaging (PWI), the time-intensity curves (TIC), relative peak intensity and outflow percentages were determined. Values were compared between treatments and with baseline.

**Results:**

Pre-treatment prominences were comparable between MRI and ultrasound (mean absolute difference 0.51 mm, *p* = 0.46), but larger differences were observed post-treatment (e.g. 3 months: 0.9 mm (*p* = 0.02)). Pre-treatment PWI metrics were comparable between treatment groups. After treatment, brachytherapy patients showed favourable changes on PWI (e.g. 67% outflow reduction at 3 months, *p* < 0.01). After PBT, significant perfusion changes were observed at a later timepoint (e.g. 38% outflow reduction at 6 months, *p* = 0.01). No consistent ADC changes were observed after either treatment, e.g. a 0.11 × 10^−3^mm^2^/s increase 12 months after treatment (*p* = 0.15).

**Conclusion:**

MR-based follow-up is valuable for PBT-treated patients as favourable perfusion changes, including a reduction in outflow, can be detected before a reduction in size is apparent on ultrasound. For brachytherapy, a follow-up MRI is of less value as already 3 months post-treatment a significant size reduction can be measured on ultrasound.

**Supplementary Information:**

The online version contains supplementary material available at 10.1007/s00234-023-03166-1.

## Introduction

Uveal melanoma (UM) is a relatively rare disease, yet it is the most common primary intraocular tumour in Caucasian adults with an incidence of between 4.4 and 10 cases per million per year [1–3]. UM arise from either the iris or the ciliary body, but most commonly the choroid (85%) [4]. Current eye-preserving treatments include episcleral brachytherapy, stereotactic external beam radiotherapy and proton beam therapy (PBT) [5]. After treatment, the primary clinical metric for assessing treatment response in UM is a reduction in tumour thickness [6–8], generally called prominence, which is conventionally obtained through ocular ultrasound. Although such a reduction in tumour prominence is generally observed in the first months after brachytherapy, it can take up to a year before it is observed after PBT [9–11]. Reflecting this, a significant increase in tumour prominence on multiple subsequent ultrasound evaluations has to be observed, before it is considered indicative of treatment failure [8]. Regardless of the generally high local control rates of ocular radiotherapy, 90–95% [12, 13], patients need to wait a relatively long time before knowing that in their specific case the tumour is also responding to the treatment, which can be quite burdensome, especially since a temporarily increase in tumour prominence on ultrasound is not uncommon after PBT [10, 13].

Recent advances in ophthalmic magnetic resonance imaging (MRI) resulted in its increased clinical use for various ophthalmologic conditions, especially for the characterisation of orbital masses [14–16]. Furthermore, MRI is extensively used to evaluate the extent of retinoblastoma [17, 18], while different studies report the use of quantitative MRI biomarkers to differentiate malignant from inflammatory or benign lesions [16, 19]. For UM specifically, studies have shown that the three-dimensional tumour visualisation provided by MRI can result in a more accurate determination of the tumour geometry and extension than conventional 2D ultrasound [20–23]. As a result, MRI is increasingly used for the PBT planning in UM [24–26]. Furthermore, functional MRIs, such as perfusion-weighted imaging (PWI) and diffusion-weighted imaging (DWI), enable the assessment of specific aspects of the tumour microenvironment without the need of an intraocular biopsy [27–30]. These MRI biomarkers are reported to correlate with known histopathologic factors of poor prognosis such as monosomy 3 [28, 31, 32].

In contrast to the pre-treatment radiological evaluation of UM, studies involving MRI in the follow-up after radiotherapy are still sparse. A notable exception is the study of Foti et al. which showed the potential of DWI for the early assessment of treatment response after PBT [27]. However, this study was limited to a description of DWI parameters alone and could not be used clinically as the used echo planar imaging (EPI) technique resulted in strong susceptibility-related artefacts, as is commonly observed in EPI-based DWI of the head and neck area [27, 29, 33]. As a result, a complete description of radiological characteristics of UM after radiotherapy is still missing and the clinical value of PWI, which has proven valuable in the follow-up of various other malignancies, including breast and prostate cancer, is unknown [34, 35].

In this study, we aim to provide a radiological description of MRI, both in terms of anatomical and functional characteristics, in the follow-up of UM patients treated with either ruthenium-106 plaque brachytherapy or PBT. In addition, we will compare MRI to the conventionally used ultrasound to assess if MRI has an added value in the follow-up of these patients.

## Materials and methods

### Patients and device

This single-centre prospective cohort study was carried out according to the Code of Ethics of the World Medical Association (Declaration of Helsinki) for experiments involving humans and was approved by the local Ethics Committee. Written informed consent was obtained from all participants. Uveal melanoma was diagnosed by ocular oncologists, based on fundoscopy, fundus photography, ultrasound and fluorescein angiography.

Patients were included in the study between March 2019 and March 2021. Following national guidelines, patients with small to intermediate-sized choroidal melanomas with a tumour prominence ≤ 7 mm and basal diameters ≤ 16 mm were considered eligible for ruthenium-106 brachytherapy, while larger tumours and juxtapapillary located tumours were referred for PBT at the HollandPTC (Delft, the Netherlands) [36]. For patients treated with brachytherapy, the dose to the tumour apex was 130 Gy, with a maximum scleral dose of 1000 Gy, as described by Marinkovic et al. [37]. The patients treated with PBT received 4 fractions of 15 Gy [38].

Patients were invited to participate in the study after diagnosis. Participants underwent an MRI and ultrasound exam at four different timepoints: before treatment and at the clinical follow-up visits at approximately 3 months, 6 months and 12 months after treatment. For brachytherapy-treated patients, all MRIs were performed in the context of this study, while for PBT-treated patients, the pre-treatment and 3-month follow-up MRIs were acquired as part of standard clinical care. All follow-up MRIs were performed during a regular clinical visit to the Department of Ophthalmology. Ultrasound measurements, performed by an ocular oncologist with an Aviso (Quantel Medical—Lumibird, Cournon-d'Auvergne, France) with a 15-MHz probe for B-scan imaging of posterior lesions and a 50-MHz probe for ultrasound biomicroscopy (UBM) of anterior lesions, were obtained from the patients’ medical files, as this is currently the clinically used method to determine the tumour size.

MRIs were performed as previously described by Ferreira et al. [28, 29]. In short, all participants were scanned with a 3-T Ingenia MRI (Philips Healthcare, Best, the Netherlands) using a 4.7-cm local receive coil (Philips). The scan protocol, as outlined in Table 1, contained three-dimensional (3D) volumetric sequences to assess tumour localization and dimensions. Two-dimensional (2D) sequences, with an increased in-plane resolution, were used to assess potential involvement of adjacent anatomical structures. In addition to T1-, T2- and contrast-enhanced T1 (T1Gd)-weighted images, diffusion- and perfusion-weighted images were acquired. For the DWI, a diffusion weighting 800 s/mm^2^ was used in combination with a turbo spin echo readout to prevent the susceptibility artefacts observed in EPI-based imaging of the orbit [29]. For the PWI, 0.1 mmol/kg bodyweight gadoterate meglumine (Gd) (Dotarem®, Guerbet Diagnostic Imaging, Villepinte, France, or Clariscan®, GE Healthcare, IL, USA) was administered with a power injector.

**Table 1:** Scan sequence parameters

Scan name	Acquisition voxel size (mm^3^)	FOV (mm^3^)	Echo train length	TE (ms)/TR (ms)	NSA	Additional parameters	Scan duration (mm:ss)
3D							
3DT1	0.8 × 0.8 × 0.8	80 × 80 × 40	14	26/400	1		2:07
3DT2 SPIR	0.8 × 0.8 × 0.8	80 × 80 × 40	117	305/2500	2		2:58
3DT1 SPIR Gd	0.8 × 0.8 × 0.8	80 × 80 × 40	14	26/400	1		2:07
2D							
MST1	0.5 × 0.5 × 2.0	100 × 100 × 24	6	8/718	1		1:16
MST2	0.4 × 0.4 × 2.0	100 × 100 × 24	17	90/1331	2		1:25
MST1 SPIR Gd	0.5 × 0.5 × 2.0	100 × 100 × 24	6	8/718	1		1:16
Functional scans							
DWI	1.25 × 1.4 × 2.4	100 × 100 × 22	Single shot	50/1555	5	*B* = 0, 800 s/mm^2^	1:33
PWI	1.25 × 1.5 × 1.5	80 × 80 × 32	88	2.3/4.5	1	2 s/dynamic TWIST	4:20

Abbreviations: 3D, three-dimensional; 2D, two-dimensional; SPIR, spectral presaturation with inversion recovery; Gd, gadolinium; MS, multi-slice; DWI, diffusion- weighted imaging; PWI, perfusion- weighted imaging; FOV, field-of-view; TE, echo time; TR, repetition time; NSA, number of signal averages; B, b-value; TWIST, time-resolved angiography with stochastic trajectories

All scans used spin echo sequences, except the PWI, which used a gradient echo sequence

### Evaluation of MR images

A radiological evaluation of all MR images was performed in Sectra IDS7 (Sectra AB, Linköping, Sweden, version 21.2) and IntelliSpace Portal (Philips Healthcare, Best, The Netherlands, version 10.1) by TGF, a neuroradiologist with 25 years of experience. At all timepoints, the tumour prominence including the sclera, apparent diffusion coefficient (ADC), time-intensity curve (TIC), quantitative PWI characteristics and presence of retinal detachment (RD) were determined on the MR images [29].

Tumour prominences were measured by TGF and JWB, an ocular imaging expert with 10 years of experience. DWI and PWI ROIs were drawn by TGF and validated by JWB. Quantitative PWI characteristics were measured by MT and JWB. Discrepancies between readers were resolved by consensus. Ultrasound measurements were performed by a single observer.

Tumour prominence was preferably determined on 3DT1Gd as its isotropic resolution enables an accurate determination of the tumour dimensions and because tumour is well differentiated from retinal detachment on these images [29]. PWI scans were evaluated both qualitatively in terms of TIC type and quantitively in terms of relative peak signal intensity and outflow percentage. Outflow percentage was defined as the relative difference between the peak intensity and signal 2 min after peak (Supplemental Fig. 1) [28]. For PWI, 2D region-of-interests (ROI) were drawn, and in the case of multiple tumour components, the ROI with the strongest outflow and/or enhancement was chosen. Qualitatively, TICs were classified as washout, plateau or progressive as earlier described by Ferreira et al. [28, 39]. Additionally, time-to-peak, peak enhancement and outflow percentage were quantified (Supplement Fig. 1) [28]. In heterogenous tumours with multiple components, the component chosen at the pre-treatment timepoint was used for all subsequent timepoints. For DWI, a representative ROI was drawn in the enhancing part of the tumour to obtain tumour ADC. For large tumours, a weighted average of the ROIs from multiple slices was used. Small tumours with a prominence < 3 mm were excluded, as these can provide unreliable ADC measurements due to partial volume effects [28].

### Statistical analysis

All statistical analyses were performed using Python (version 3.7, Python Software Foundation, DE, USA) and the SciPy package (version 1.8.0). *p*-values equal to or below 0.05 were considered significant. All quantitative MRI measurements were compared using Student’s *T*-tests. In addition, the tumour prominences between ultrasound and MRI were compared using paired *T*-tests. At each timepoint, comparisons were made between brachytherapy and PBT groups. Additionally, comparisons were made between baseline and follow-up timepoints.

At each follow-up timepoint, the tumour prominence, both on MRI and ultrasound, ADC value, peak intensity and perfusion outflow percentage were compared with baseline to assess if there were changes indicative of therapy response. Similar to the study of Foti et al. [27], and corresponding to the common practice in ocular oncology [8, 10, 13], we used a reduction in tumour prominence as the primary measure of treatment response. Given the 0.3-mm interobserver standard deviation of ultrasonic prominence measurements [40], a 95% CI reduction in tumour prominence, corresponding to 0.6 mm, was considered an indicator of response for both MRI and ultrasound. For PWI, a perfusion outflow decrease of 5% could qualitatively still be accurately distinguished and (Supplement Fig. 2); therefore, a ≥ 5% decrease in perfusion outflow was considered a functional sign of therapy response on MRI. As the relative peak intensity varies depending on the degree of tumour pigmentation, this metric was not included as a measure indicative of therapy response [30].

## Results

### Patient baseline characteristics

**Fig. 1:**
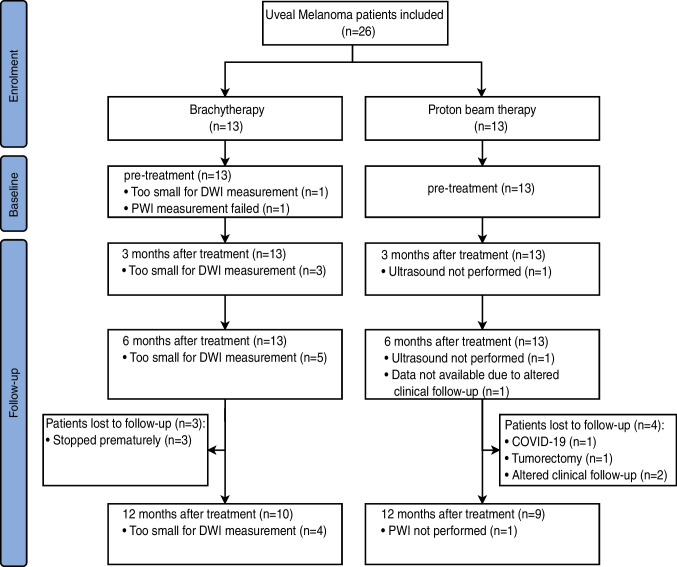
Patient inclusion flowchart

**Fig. 2:**
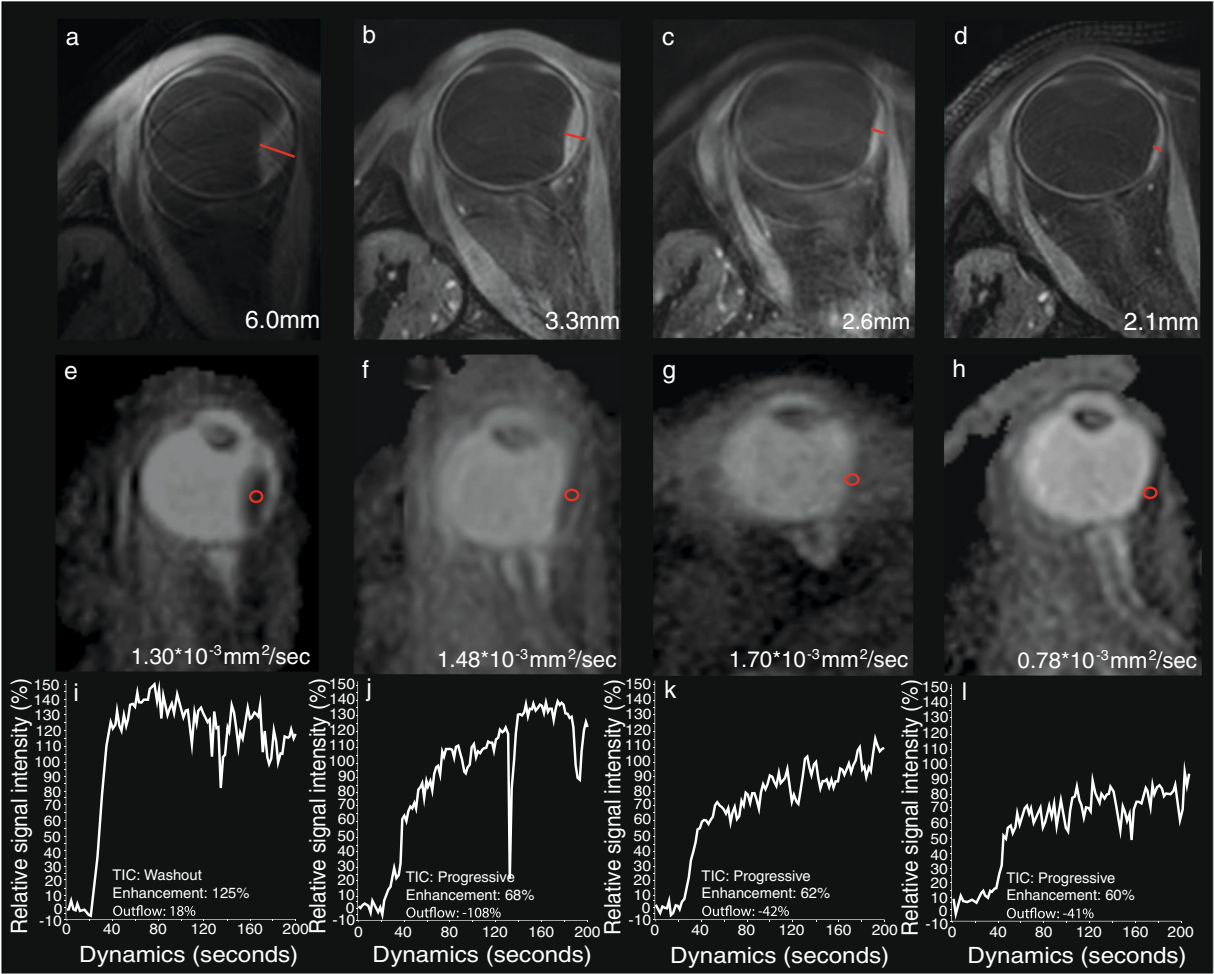
MRI of a UM of the right eye (**a**,** e**, **i**) before, and (**b**,** f**,** j**) 3, (**c**,** g**,** k**) 7 and (**d**,** h**,** l**) 13 months after ruthenium plaque brachytherapy. Favourable evolution of the UM was observed in terms of size (red lines in **a**–**d**), ADC value and TIC at dynamic contrast-enhanced MR perfusion (DCE). **a**–**d** Axial oblique multi-slice (MS) turbo spin echo (TSE) contrast-enhanced T1 with fat signal suppression. Progressive decrease in size of the UM, indicated by red lines, already noticed 3 months after radiotherapy. **e**–**h** Axial oblique ADC. Progressive increase of the UM ADC value, except 13 months after radiotherapy where there is a decrease. Regions of interests used to derive the ADC values are delineated in red circles. **i**–**l** DCE TICs. Progression of the initial washout TIC profile into a plateau and progressive TIC, corresponding to a lower (more negative) outflow percentage

**Fig. 3:**
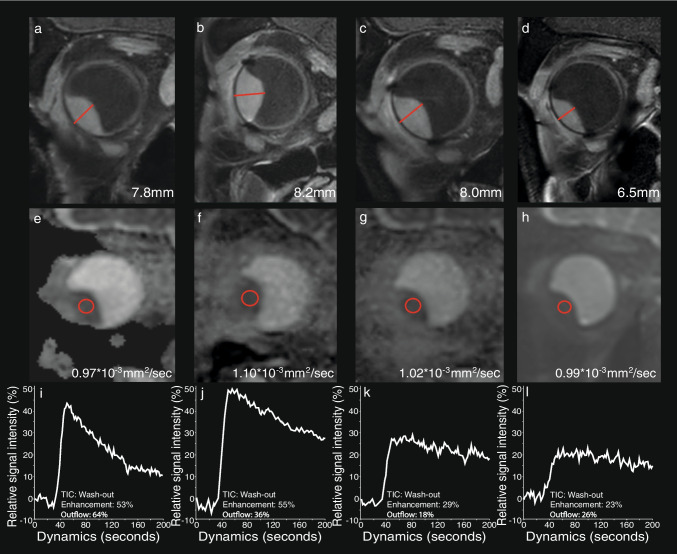
MRI of a UM of the right eye (**a**,** e**, **i**) before, and (**b**,** f**,** j**) 2, (**c**,** g**,** k**) 5 and (**d**,** h**, **l**) 11 months after PBT. Pseudo progression of the UM 2 months after radiotherapy: slight increase in size, indicated by red lines (**a**–**d**), but what seems a more favourable TIC in terms of perfusion outflow reduction at DCE MR Perfusion. **a**–**d** Coronal oblique MS TSE contrast-enhanced T1 with fat signal suppression. Slight increase in size of the UM 2 months after radiotherapy followed by a slow progressive decrease of its size. **e**–**h** Coronal oblique ADC. Slight increase of the UM ADC value at 2 months which remained stable. **i**–**l** DCE TIC. Favourable perfusion characteristics after PBT, with progressive smaller peak intensity and outflow percentage. ROIs used to derive the ADC values are delineated in red circles

A total of 26 UM patients were enrolled in the study, 13 patients treated with PBT and 13 with ruthenium brachytherapy. Seven patients were lost during follow-up and did not reach the 1-year timepoint: three treated with brachytherapy and four with PBT. Four PBT patients did not have an MRI scan after the 6-month timepoint due to an altered clinical follow-up, a tumorectomy due to an exudative retinal detachment, and COVID-19 infection (Fig. 1). Three brachytherapy patients prematurely stopped participating after the 6-month follow-up out of own volition. Additionally, one PBT patient did not have a 6-month examination due to an altered clinical follow-up, but did get an examination 1 year post-treatment. MR images of a representative PBT and brachytherapy patient are shown in Figs. 2 and 3.

Patient and tumour characteristics are listed in Table 2. Mean UM prominence measured on ultrasound was 4.9 mm (± 1.3) for brachytherapy and 8.8 mm (± 2.4) for PBT patients. On perfusion-weighted MRI, the TICs were comparable between groups as 11/13 (85%) brachytherapy and 8/12 (67%) PBT patient TICs were of the washout type, while the remainder were of the plateau type. Mean ADC values and standard deviations were 1.20 × 10^−3^ mm^2^/s (± 0.20) for brachytherapy and 0.99 × 10^−3^ mm^2^/s (± 0.14) for PBT patients.

**Table 2:** Baseline characteristics

Characteristics	Patients (*n* = 26)
Sex — male	15 (58%)
Age — median (yrs)	65 (range 30–84 years)
Eye — OS	11 (42%)
US prominence — mean (SD)	6.8 mm (± 2.8 mm)
US LBD — mean (SD)	13.9 mm (± 3.0 mm)
AJCC classification*	
T1/T2/T3/T4	2 (8%)/10 (38%)/13 (50%)/1 (4%)
Brachytherapy/PBT	13 (50%)/13 (50%)
MRI to treatment — mean (days)	22 days (± 15)
Retinal detachment — yes	15 (58%)
Shape (%)	
Mushroom	4 (15%)
Dome	15 (58%)
Lentiform	7 (27%)

Data are presented as number of patients (%) for categorical variables and as mean ± SD for continuous variables

*According to the 8^th^ American Joint Committee on Cancer (AJCC) edition

### Prominence

A detailed statistical evaluation of prominence changes can be found in Supplementary data.

**Fig. 4:**
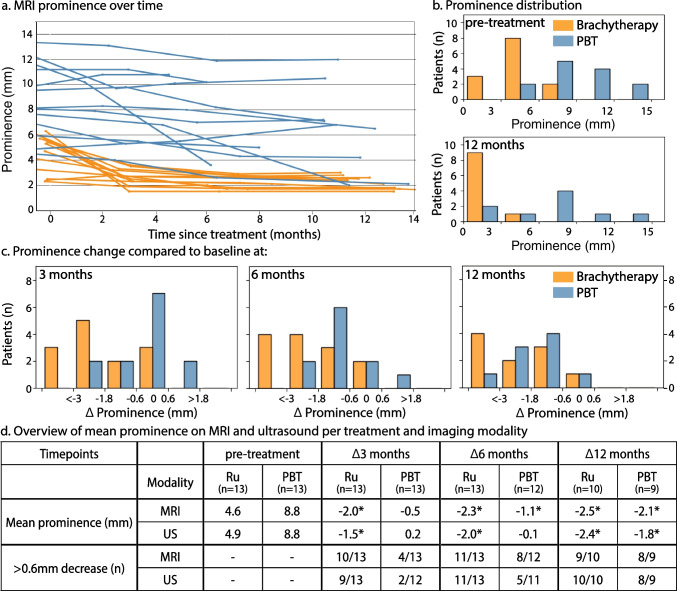
Overview of prominence regression on MRI after radiotherapy. **a** The regression of the tumour prominence measured on MRI for patients treated with brachytherapy (orange) or PBT (blue). **b** The distribution of UM measured prominence (top) before treatment and (bottom) 12 months after treatment. **c** MRI-based relative change in prominence (mm) measured at (left) 3, (middle) 6 and (right) 12 months after treatment compared to pre-treatment. **d** Table shows the comparison between prominence measurements on ultrasound and MRI at different timepoints. Significant (*p* ≤ 0.05) changes compared to pre-treatment are marked by an asterisk

Three months after treatment, the tumours treated with brachytherapy showed a significant decrease on MRI compared to baseline of 1.9 mm on average (*p* < 0.01), which was also observed on ultrasound (Fig. 4). The majority of these patients, 10/13 (77%), showed a decrease in prominence of at least 0.6 mm, which would therefore be considered as response to treatment. Only one UM patient treated with brachytherapy showed an increase in prominence on MRI after treatment. This, however, involved a very flat tumour, which showed a, non-significant, 0.4-mm increase at the 3-month timepoint, followed by a significant 0.8-mm reduction 12 months after treatment.

At 6 months and 1 year after brachytherapy, a further significant decrease in prominence was observed on MRI, with an average reduction of 2.3 mm (*p* < 0.01) and 2.5 mm (*p* < 0.01) respectively. None of these patients showed an increase in prominence compared to the previous MRI. At the 1-year follow-up, 9/10 (90%) patients showed a tumour reduction of at least 0.6 mm and the average prominence of the remaining lesion including sclera was 2.2 mm.

For PBT-treated patients, a non-significant decrease in prominence, on average 0.5 mm compared to baseline (*p* = 0.11), was observed on MRI 3 months after treatment. Four of 13 (31%) of these patients showed a decrease in tumour prominence of at least 0.6 mm, while five patients showed an increase in tumour prominence of up to 1.1 mm. For some of the patients whose prominence remained unchanged 3 months after PT, the three-dimensional evaluations of the MR images did show qualitatively a slight reduction in overall tumour volume. At 6 months, an average significant decrease of 1.1 mm (*p* = 0.03) compared to pre-treatment was observed, with 8/11 (73%) patients showing a tumour reduction of at least 0.6 mm. Compared to the 3-month MRI, none of the patients showed an increase in prominence. At the 1-year follow-up, a larger decrease in prominence was found at 2.1 mm on average (*p* < 0.01), but a similar percentage of patients, 8/9 (89%) patients, showed a prominence reduction of more than 0.6 mm. The average prominence 1 year after proton therapy was 6.5 mm.

US- and MRI-based prominence measurements did not differ statistically before treatment (mean absolute difference 0.51 mm, *p* = 0.46) (Supplement Table. 2). At 3-month and 6-month follow-ups, however, the ultrasound prominence measurements differed significantly from MRI (mean absolute difference: 0.9 mm; *p* < 0.01 and 0.7 mm; *p* < 0.01, respectively). At these timepoints, ultrasound measured on average larger prominences than MRI (Supplement Table. 1).

**Fig. 5:**
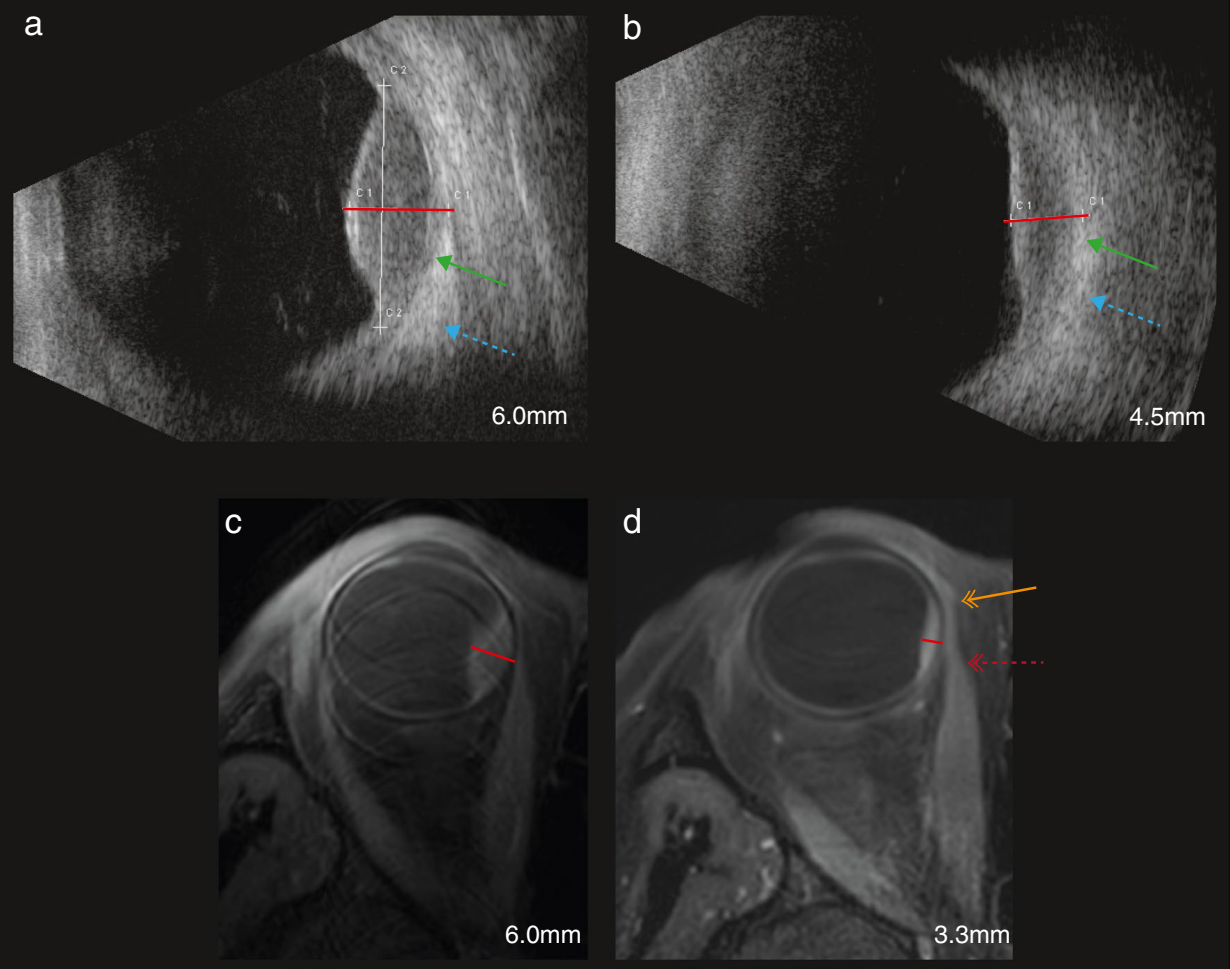
**a**,** b** Ultrasound and **c**,** d** MRI of a UM of the right eye **a**,** c** before and **b**, **d** 3 months after ruthenium plaque brachytherapy. Post-treatment changes, accounting for less accurate ultrasound measurements of the UM after radiotherapy, but not interfering with the MR measurements. The tumour prominence measured 1.2 mm thicker on ultrasound as compared to MRI (red lines) after brachytherapy, which could likely be attributed to structural changes of the extra-ocular tissue due to radiation reactions or due to the temporary surgical detachment of the extra-ocular muscle adjacent to the tumour needed to facilitate the placement of the ruthenium applicator. **a**, **b** Ultrasound images with the identification of the limits of the UM, of the sclera (green arrow) and of the medial rectus (blue dashed arrow) being easy before and difficult after radiotherapy. **c**, **d** Axial oblique MS TSE contrast-enhanced T1 with fat signal suppression. Although there is post-radiotherapy peri-scleral enhancement (orange arrow with double chevron), and thickening of the tendon of the medial rectus due to its intraoperative detachment during the ruthenium plaque insertion (red dashed arrow with double chevron), the limits of the tumour and of the sclera remain clearly visible, allowing accurate measurements

A joint retrospective analysis of the ultrasound and MR images of these patients by a neuroradiologist (TGF), ocular oncologist (MM) and ocular imaging expert (JWB) showed a low contrast between the outer scleral layer and extra-ocular structures on the ultrasound images, while on MRI the sclera as well as post-radiotherapy effects could be discerned (Fig. 5d). As a result, it appeared that on ultrasound the outer scleral border was assumed to be located more distant from the mass, resulting in the erroneous inclusion the adjacent ocular muscles or structures on ultrasonic prominence measurement (Fig. 5b).

### PWI

In two MRI exams, no contrast was administered, resulting in missing PWI data of one pre-treatment and one 1-year follow-up exam. The lack of the pre-treatment PWI scan prevented the calculation of relative differences in subsequent PWI scans of this patient.

**Fig. 6:**
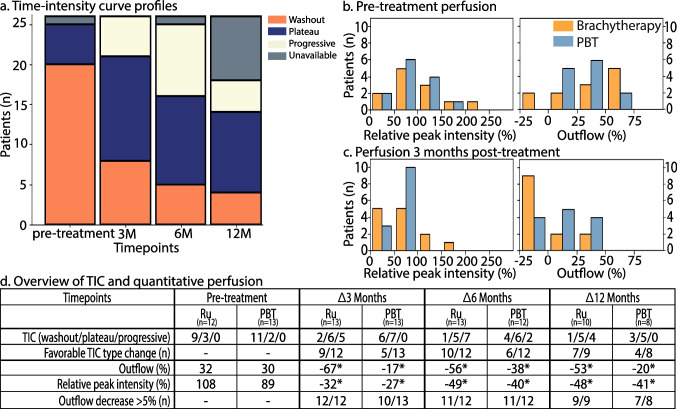
Overview of PWI metrics before and after ocular radiotherapy. **a** The TIC profiles show a gradual progression from primarily washout to plateau/progressive curves. **b** Pre-treatment and **c** 3-month post-treatment (left) relative peak intensity and (right) outflow percentage for brachytherapy (orange) and PBT (blue) patients. **d** Summary of the distribution in TIC profile, outflow percentage, relative peak intensity percentage and treatment response at all timepoints for the different treatments. Significant (*p* ≤ 0.05) outflow percentage and relative peak intensity changes are marked by an asterisk

Overall, for the majority of the patients, the TIC profiles showed less enhancement and less outflow after treatment, an evolution which in other tumour sites would be considered favourable (Fig. 6).

For brachytherapy-treated patients, a qualitative assessment of perfusion data at 3 months after treatment showed a favourable change in TIC type in 9/12 (75%) patients. Quantitative analysis showed that the peak intensity and outflow percentage both significantly decreased by respectively 32% and 67% (*p* < 0.02) compared to baseline. Furthermore, in 12/12 (100%) of these patients, a reduction of at least 5% in outflow compared to baseline was observed. These perfusion changes continued progressively over the course of the first year. At the 1-year follow-up, the average peak intensity and outflow percentage had decreased significantly compared to baseline by respectively 48% and 53% (*p* ≤ 0.05) and only 1/10 (10%) patients showed a washout TIC.

At 3 months after proton beam therapy, 5/13 (38%) patients showed a more favourable TIC. Average peak intensity and outflow percentage decreased significantly by respectively 26% and 17% (*p* < 0.01) compared to baseline, and in 10/13 (77%) patients, at least a 5% reduction in outflow was observed. In one of the patients without a 5% reduction in outflow, eye motion during the PWI scan at this timepoint was missed during the clinical evaluation, which resulted in an erroneous low outflow percentage. After correction of this eye motion, this patient did show a 9% reduction in outflow. For the remaining two patients, a significant decrease in relative peak intensity of at least 30% was observed, which overshadowed the reduction in outflow (Supplement Fig. 1). Perfusion changes continued gradually in the following timepoints showing a further reduction in peak intensity and outflow percentage. At the 1-year follow-up, average peak intensity and outflow percentage decreased significantly compared to baseline by respectively 41% and 20% (*p* ≤ 0.02) and either a washout (37%) or plateau (63%) TIC was observed.

### DWI

From 3 months onwards for one patient and from 6 months onward for an additional four patients, no reliable ADC measurements could be obtained as the prominence was < 3 mm. DWI data from these timepoints were excluded from analysis.

**Fig. 7:**
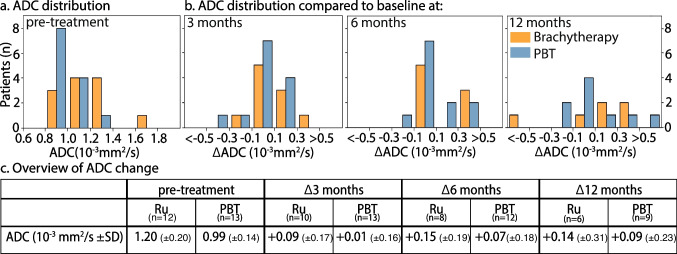
Overview of DWI data before and after radiotherapy. **a** Before treatment, the brachytherapy (orange) patients showed a significant (*p* = 0.01) higher ADC compared to the PBT (blue)-treated patients. **b** ADC changes (left) 3, (middle) 6 and (right) 12 months after treatment compared to pre-treatment. **c** Table with mean ADC values and standard deviations at different timepoints. Significant (*p* ≤ 0.05) differences compared to baseline are marked by an asterisk

Regardless of treatment, large variations in ADC changes were found between subjects, e.g. at 3-month follow-up ADC differences compared to baseline ranged from − 0.36 to + 0.45 × 10^−3^ mm^2^/s. At 3 months, a mean ADC increase of 0.04 × 10^−3^ mm^2^/s compared to baseline was observed which was not significant (*p* = 0.26) (Fig. 7c). At 6 months, mean ADC increased by 0.10 × 10^−3^ mm^2^/s which was significant (*p* = 0.03). At 1-year follow-up, however, a mean ADC increase of 0.11 × 10^−3^ mm^2^/s was not found to be significant (*p* = 0.15). For the detailed evaluation, refer to Supplementary data.

### Combined assessment

**Fig. 8:**
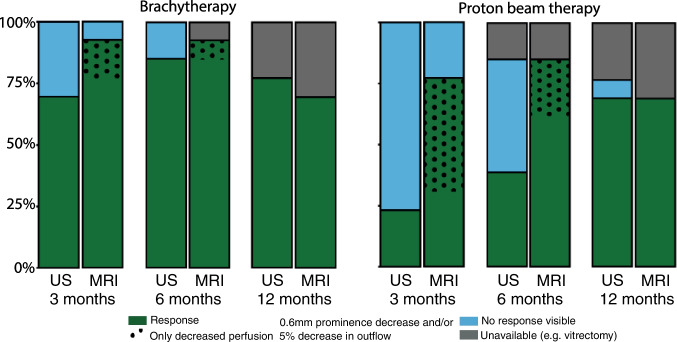
A side-by-side comparison of treatment response of (left) brachytherapy and (right) PBT-treated patients, based on MRI and ultrasound at each timepoint. Treatment response in terms of tumour prominence was defined as a decrease in tumour prominence of more than 0.6 mm. In addition, a reduction in outflow percentage of at least 5% on PWI was also considered a sign of treatment response on MRI. At 3 months, 69% of brachytherapy patients showed treatment response on ultrasound as compared to 92% patients on MRI. For PBT patients, however, only 23% of patients showed treatment response on ultrasound while this was 77% on MRI. Furthermore, 60% of those patients who showed treatment response on MRI were based on favourable PWI changes, when no prominence decrease was yet apparent

When using size alone as an indicator of treatment response, MRI and ultrasound could show a more than 0.6-mm decrease in prominence in approximately the same number of patients, regardless of treatment and timepoint, e.g. 4/13 (31%) on MRI vs 3/13 (23%) on ultrasound 3 months after PBT. When, however, a decrease in outflow is included as an additional indicator of treatment response, MRI shows earlier signs of treatment response than ultrasound (Fig. 8). Three months and six months after PBT, for example, MRI shows signs of response in respectively 10/13 (77%) and 11/11 (100%) of patients, whereas ultrasound only showed this in respectively 3/13 (23%) and 5/11 (45%) of these patients. At the 1-year timepoint, no such difference was observed for both the brachytherapy and PBT patients, as more than 85% of the patients showed a sufficient reduction in prominence.

## Discussion

### General observations

Overall, on MRI, two distinct types of evolution of the UM after radiotherapy were observed. In the first scenario, primarily observed in patients treated with ruthenium brachytherapy, a progressive decrease in size of tumour is observed which is accompanied by progressively more favourable perfusion characteristics. A representative example of this scenario is shown in Fig. 2. In these patients, a clear reduction in tumour prominence was apparent at the 3-month timepoint. Additionally, favourable perfusion changes were visible in the majority of the patient’s TICs, such as the progression from an initial washout TIC into a plateau TIC. Compared to pre-treatment, the DWI at follow-up showed no clear overall changes in these patients.

In the second scenario, shown in Fig. 3, a much slower change was observed on all aspects. This scenario corresponded generally to the patients with a larger tumour who were treated with PBT. In these patients, no significant reduction in prominence was observed at the first timepoint after therapy, and in some patients, even a small increase in prominence was measured. In most of these patients, the TIC type did not change at the first follow-up visit, but often more subtle favourable perfusion changes were visible, such as a decreased washout. At the later timepoints, changes could more clearly be observed, although at the 12-month timepoint still a significant part of the tumour mass was present and 3/8 (38%) patients retained a washout TIC. Similar to the other scenario, no clear pattern was observed on DWI. In these patients, an increase in retinal detachment was often observed early after PBT.

The observed slower evolution in UM patients treated with PBT compared to brachytherapy is in agreement with earlier studies that assessed the prominence reduction over time with ultrasound [11]. This slower evolution could potentially be attributed to the significantly higher dose that is used in ocular brachytherapy compared to PBT [10, 11, 37, 38]. Although a larger study with similarly sized tumours for both treatments is needed to further assess the origin of this difference, the slower change in perfusion metrics in the PBT group confirms the suggestion that the initial size of the lesion is not the primary factor responsible for this difference.

### Tumour dimensions

In current clinical practice, a reduction in tumour prominence on 2D ultrasound cross sections is the most commonly used criterion to assess treatment response in UM [10, 13]. On both MRI and ultrasound, the brachytherapy-treated tumours showed a rapid decrease in prominence. For the PBT patients, however, a much slower progression was observed with the majority of patients not yet showing a decrease in prominence 3 months after treatment. Some patients even showed a slight increase in prominence, so-called pseudo progression [41], which reduced in subsequent timepoints. Additionally, we did note that in some PBT patients with no change in tumour prominence, on MRI, the tumour volume visually appeared to be reduced. Such a volumetric assessment of the response to treatment warrants further study.

The pre-treatment MRI- and ultrasound-based prominence measurements were generally in agreement, as has also been reported in other studies [20, 23, 28, 42]. In some patients, MRI’s three-dimensional tumour visualisation allowed for a more reproducible comparison of the tumour prominence, as exactly the same plane could be used for measurements at all timepoints. Similar to earlier studies, the largest prominence differences between MRI and ultrasound were found in anteriorly located tumours [23, 28], for which MRI can generally be considered more accurate [22, 23]. Additionally, we observed larger differences between both modalities 3 and 6 months after radiotherapy, with ultrasound providing on average 0.67 mm larger prominences. On the follow-up MR images of some of these patients, we observed changes to the tissues directly adjacent to the tumour, such as hyperintensities on T2 or enhancement after contrast administration (Fig. 5). In some cases, these changes could be directly attributed to the treatment, e.g. the temporary detachment of the extra-ocular muscle to facilitate the placement of the ruthenium applicator, or to the 2.5-mm margins used in PBT. On the corresponding ultrasound images, however, only a loss of contrast between the sclera and extra-ocular structures was observed, and parts of these structures were erroneously included as sclera in the prominence measurement. As these effects reduce over time, the differences between ultrasound and MRI reduced were statistically non-significant 12 months post-treatment (Supplement Table. 2, *p* = 0.29). For the brachytherapy patients, this time course is in agreement with the surgical experience of a changed, more edematous and fragile, structure of the extra-ocular muscle in the first months after surgically detaching it from the sclera.

### DWI

We observed a minimal increase in average ADC over time in both the PBT and brachytherapy cohort, e.g. 0.11 × 10^−3^ mm^2^ (± 0.27) over a 1-year period. Although generally an increase in ADC is considered a sign of therapy response [19, 27, 35], 65% of the patients showed a decrease in ADC at one or more timepoints. Conversely to the study of Foti et al., we found no significant increase in the average ADC 3 months after treatment [27], and our data suggests that DWI cannot be used to assess treatment response after ocular radiotherapy. We hypothesise that the observed decrease in ADC could be attributed to radiation-induced microscopic areas of necrosis or blood [43], which, according to a more recent study of Foti et al., are too small to be visible on MRI [44].

### PWI

In contrast to DWI, PWI showed a consistent, favourable, progression after radiotherapy at the individual patient level, such as a progressive TIC type or a slower washout rate. Three months post-treatment, these changes were already visible in 85% of the patients. As at this timepoint a reduction in size was not yet apparent in 31% of these patients, PWI could be considered an early measure of treatment response. Due to the strong perfusion changes after treatment, relatively simple metrics, such as peak enhancement and outflow percentage [28], were generally sufficient to detect changes for an individual patient. For example, although nearly a fourth of the UM patients retained a washout TIC 1 year post-treatment, the TICs of these patients did show a decrease of both the peak intensity and outflow percentage. However, we noted that the reduction in peak enhancement can mask a reduction in outflow as it confounds the used outflow percentage. It would therefore be valuable to perform pharmacokinetic modelling on these PWI data [30, 45, 46], as this separates these changes and thereby more accurately quantify changes in the tissue’s perfusion.

### Clinical recommendations

Although this study included a relatively small cohort of 26 patients, the progression of the radiological characteristics after radiotherapy matches the general trends, such as a gradual decrease in tumour size and favourable perfusion changes, observed in other tumours such as gliomas, breast and prostate cancers [34, 47, 48]. Furthermore, a consistent, but therapy dependent, evolution of tumour characteristics was observed across the individual patients. Although we therefore judge these findings as representative, we do acknowledge that indications for different types of ocular radiotherapy vary between institutes [38]. Institutes that do not offer brachytherapy, for example, might observe slightly different results as they would also treat smaller tumours with PBT.

For patients treated with ruthenium-106 brachytherapy, the added value of a follow-up MRI is limited. These tumours show a rapid, > 1.9 mm on average, prominence reduction 3 months after treatment, which can reliably be detected on ultrasound in the vast majority of patients (Fig. 8).

In patients treated with PBT, however, MRI proved to be valuable in the early assessment of treatment response. As in the first months after PBT these tumours showed little to no change on ultrasound [10], MRI’s more accurate and reproducible prominence measurements are less likely to falsely identify tumour growth [13]. However, MRI’s main added value is the detection of perfusion changes that precede a reduction in prominence. As a result, 3 months post-treatment, MRI can provide a confirmation of treatment response in 85% of the patients, whereas ultrasound could only show a reduction in size in 46% (Fig. 7). These favourable perfusion changes, indicating favourable changes at the microvascular level, can aid treating physicians in making more well-informed decisions such as opting to maintain a wait-and-see policy instead of proceding with a surgical intervention, e.g. an endoresection or enucleation, when no reduction in prominence is yet apparent. As these changes could be reliably detected 3 months post-treatment, we would recommend to combine the follow-up MRI with the 3- to 6-month follow-up visit to the ophthalmologist. As the majority of patients still do not yet show a progressive TIC at this time, a pre-treatment MRI is required to assess the more subtle changes in perfusion. Although such an MRI is currently not performed in all centres, MRI is increasingly being used for ocular proton therapy planning [22, 25, 32].

Independent of type of treatment, MRI can be relevant for patients who receive a vitrectomy to resolve a retinal detachment, a common complication in ocular oncology. As the used silicon oil tamponade prevents subsequent ultrasound imaging, MRI might be the only modality to assess tumour dimensions [49].

In this study, the conventional evaluation of the response to treatment was limited to a reduction in prominence as obtained by ultrasound. In clinical practice, however, fundoscopy can provide additional, qualitative, imaging clues of therapy response, such an atrophic appearance. Although we acknowledge that these changes can clinically be a valuable sign when no size reduction is evident on ultrasound, they are limited as they can only assess the lesion’s ventral surface and lack the quantitative metrics as provided by functional MRI. An additional limitation of using a reduction in prominence as the primary metric of treatment response is that it predominantly assesses the central part of the tumour, thereby potentially missing growth at a more peripheral part of the tumour. Given the heterogeneity of UM, also in terms of perfusion, an analysis which separately evaluates these distinct tumour parts might be more appropriate [30]. Furthermore, in accordance with the high rates of local control of 95% and 94% for ruthenium brachytherapy and PBT respectively [36, 37], no recurrences were present in our cohort. A study with a longer follow-up is therefore warranted to determine if the observed biomarkers of therapy response are also indicative of a good response with no local recurrences on the longer term.

Overall, we conclude that MR-based follow-up is particularly valuable for patients treated with ocular proton therapy, as the inclusion of perfusion changes, such as a reduction in outflow, in the evaluation provides earlier indications of therapy response compared to ultrasound. For these patients, the inclusion of MRI might not only reduce the stressful period of uncertainty in which they do not yet know if their tumour is responding to treatment, but it could also reduce the frequency of follow-up visits due to inconclusive ultrasound exams [13]. For brachytherapy, a follow-up MRI is of less value as a significant reduction in tumour prominence could also be observed on ultrasound 3 months after treatment.

## Supplementary Information

Below is the link to the electronic supplementary material.

Supplementary file1 (PDF 384 KB)

Supplementary file2 (CSV 5 KB)

## Data Availability

Data may be available upon request from the Ophthalmology Science Committee (contact via oog.stafsecretariaat@lumc.nl) for researchers who meet the criteria for access to confidential data.
