# Supplementary material for: MR-based follow-up after brachytherapy and proton beam therapy in uveal melanoma
Source: Neuroradiology. 2023 May 30;65(8):1271–85. doi: 10.1007/s00234-023-03166-1 (PMC10227806; doi:10.1007/s00234-023-03166-1)
Supplement: Supplementary file 1 — Supplementary file1 (PDF 384 KB) [file 234_2023_3166_MOESM1_ESM.pdf]

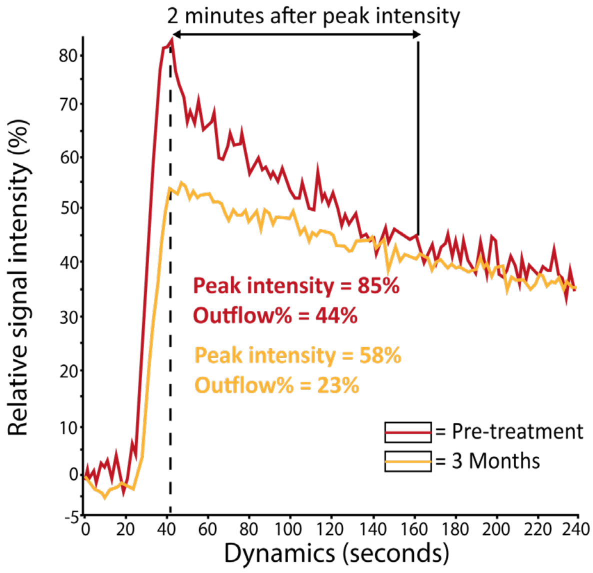

Supplemental Fig. 1 Two TICs derived from PWI of one UM patient (red) before and (orange) three months (orange) after PBT. Both TICs were classified as wash-out type curve profiles, however, the post-treatment TIC already showed a clear decrease in both relative peak intensity (-27%) and outflow percentage (-21%) indicative of biological tumour change

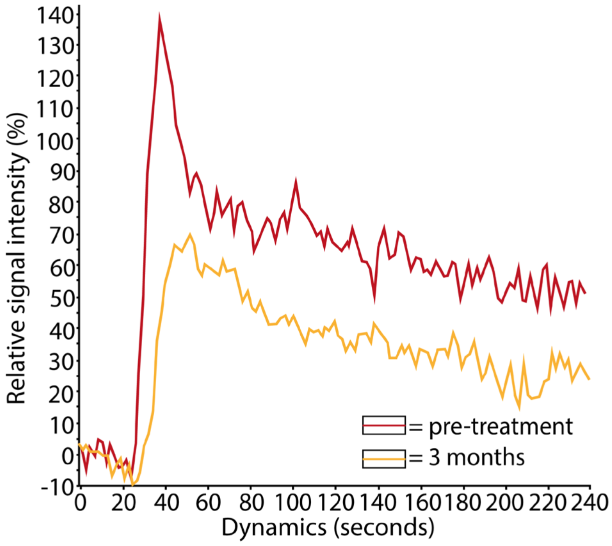

Supplemental Fig. 2 Two TICs derived from PWI of one UM patient (red) before and (orange) three months (orange) after PBT. Although, the post-treatment TIC only showed a 7% decrease in outflow percentage, qualitatively it could still be distinguished from the pre-treatment TIC

Supplemental Table 1. An overview of the paired t-tests performed for the prominence measured on MRI between different treatments at three, six and 12 month follow-up. Significant values (p≤0.05) are marked by an asterisk

Modality	Treatment	Measurements compared to baseline at:	Mean prominence (mm)	p-value
MRI	Brachytherapy	3 months	2.63	<0.01*
		6 months	2.26	<0.01*
		12 months	2.20	<0.01*
	PBT	3 months	8.31	0.11
		6 months	7.27	0.03*
		12 months	6.49	<0.01*
	Combined	3 months	5.47	<0.01*
		6 months	4.66	<0.01*
		12 months	4.23	<0.01*

Supplemental Table 2. An overview of the paired t-tests performed for the prominence measured on ultrasound between different treatments at three, six and 12 month follow-up. Significant values (p≤0.05) are marked by an asterisk

Modality	Treatment	Measurements compared to baseline at:	Mean prominence (mm)	p-value
Ultrasound	Brachytherapy	3 months	3.35	<0.01*
		6 months	2.86	<0.01*
		12 months	2.51	<0.01*
	PBT	3 months	9.20	0.40
		6 months	8.44	0.81
		12 months	6.74	<0.01*
	Combined	3 months	6.16	0.02*
		6 months	5.42	<0.01*
		12 months	4.51	<0.01*

Supplemental Table 3. An overview of the paired t-tests performed for the prominence measured on ultrasound and MRI between different treatments at three, six and 12 month follow-up. Significant values (p≤0.05) are marked by an asterisk

Modality	Treatment	Measurements compared between modalities at:	Mean absolute difference(mm)	p-value
Ultrasound vs MRI	Brachytherapy	pre-treatment	0.49	0.08
		3 months	0.75	0.01*
		6 months	0.65	<0.01*
		12 months	0.41	0.01*
	PBT	pre-treatment	0.50	0.74
		3 months	1.03	0.10
		6 months	0.85	0.02*
		12 months	0.71	0.34
	Combined	pre-treatment	0.49	0.46
		3 months	0.88	<0.01*
		6 months	0.74	<0.01*
		12 months	0.55	0.06

Supplemental Table 4. An overview of the statistical tests performed for DWI ADC values between between different treatments at three, six and 12 month follow-up. Significant values ($p \leq 0.05$) are marked by an asterisk

Modality	Treatment	Measurements compared to baseline at:	Mean ADC value (*10^{-3} mm^2/sec)	p-value
MRI (DWI)	Brachytherapy	3 months	1.28	0.15
		6 months	1.36	0.08
		12 months	1.33	0.37
	PBT	3 months	0.99	0.91
		6 months	1.07	0.23
		12 months	1.08	0.31
	Combined	3 months	1.12	0.26
		6 months	1.19	0.03*
		12 months	1.18	0.15

Supplemental Table 5. An overview of the statistical tests performed for PWI **(a)** relative peak intensity and **(b)** outflow percentage between between different treatments at three, six and 12 month follow-up. Significant values ($p \leq 0.05$) are marked by an asterisk

a

Modality	Treatment	Measurements compared to baseline at:	Mean relative peak intensity (%)	p-value
MRI (PWI)	Brachytherapy	3 months	73.1	0.02*
		6 months	58.3	0.01*
		12 months	64.6	0.05*
	PBT	3 months	62.6	0.01*
		6 months	49.6	<0.01*
		12 months	45.8	0.02*
	Combined	3 months	67.8	<0.01*
		6 months	54.2	<0.01*
		12 months	56.2	<0.01*

b

Modality	Treatment	Measurements compared to baseline at:	Mean outflow percentage (%)	p-value
MRI (PWI)	Brachytherapy	3 months	-32.0	<0.01*
		6 months	-21.9	<0.01*
		12 months	-26.9	<0.01*
	PBT	3 months	12.7	0.01*
		6 months	-7.3	<0.01*
		12 months	11.6	0.01*
	Combined	3 months	-9.6	<0.01*
		6 months	-14.9	<0.01*
		12 months	-9.8	<0.01*
